# PIWI proteins and piRNAs: key regulators of stem cell biology

**DOI:** 10.3389/fcell.2025.1540313

**Published:** 2025-02-06

**Authors:** Fernando Claro-Linares, Patricia Rojas-Ríos

**Affiliations:** Departamento de Genética, Facultad de Biología, Universidad de Sevilla, Sevilla, Spain

**Keywords:** PIWI proteins, piRNAs, germline stem cells, *Drosophila*, mRNA regulation

## Abstract

In this mini review, we discussed the functional roles of PIWI proteins and their associated small RNAs, piRNAs, in regulating gene expression within stem cell biology. Guided by piRNAs, these proteins transcriptionally and post-transcriptionally repress transposons using mechanisms such as the ping-pong amplification cycle and phasing to protect germline genomes. Initially identified in *Drosophila melanogaster*, the piRNA pathway regulate germline stem cell self-renewal and differentiation via cell-autonomous and non-cell-autonomous mechanisms. Precisely, in GSCs, PIWI proteins and piRNAs regulate gene expression by modulating chromatin states and directly influencing mRNA translation. For instance, the PIWI protein Aubergine loaded with piRNAs promotes and represses translation of certain mRNAs to balance self-renewal and differentiation. Thus, the piRNA pathway exhibits dual regulatory roles in mRNA stability and translation, highlighting its context-dependent functions. Moreover, PIWI proteins are essential in somatic stem cells to support the regenerative capacity of highly regenerative species, such as planarians. Similarly, in *Drosophila* intestinal stem cells, the PIWI protein Piwi regulates metabolic pathways and genome integrity, impacting longevity and gut homeostasis. In this case, piRNAs appear absent in the gut, suggesting piRNA-independent regulatory mechanisms. Together, PIWI proteins and piRNAs demonstrate evolutionary conservation in stem cell regulation, integrating TE silencing and gene expression regulation at chromatin and mRNA levels in somatic and germline lineages. Beyond their canonical roles, emerging evidence reveal their broader significance in maintaining stem cell properties and organismal health under physiological and pathological conditions.

## Introduction

The *piwi* (for *P*-element *i*nduced *wi*mpy testis) gene was initially identified in a genetic screen of single P-element mutants as a key regulator of Germline Stem Cell (GSC) asymmetric division in *Drosophila melanogaster* (Lin and Spradling, 1997). Several years later, a novel class of small non-coding RNAs, termed PIWI-interacting RNAs (piRNAs), was discovered in the germline of various animal species (Aravin et al., 2006; Girard et al., 2006; Grivna et al., 2006; Lau et al., 2006; Vagin et al., 2006). These RNAs were named piRNAs because they associate with the PIWI-clade subfamily of the Argonaute protein family. PIWI proteins are highly expressed in animal gonads and exhibit RNA-endonucleolytic activity guided by piRNAs. piRNAs are typically 23–31 nucleotides long. Unlike the PIWI proteins, which are highly conserved throughout evolution, piRNAs exhibit low sequence conservation across species (Özata et al., 2020). Despite this variability, the presence and biological roles of piRNAs are well conserved in the germline of many animals, where they are crucial for repressing transposable elements (TEs). TEs are parasitic and highly abundant DNA sequences capable of replicative transposition, enabling them to move to new genomic regions. Thus, the movement of TEs compromises genome integrity, and piRNAs play an essential role in protecting the genome from such damage. The mode of action of the piRNA pathway in TE silencing is either at the transcriptional or at post-transcriptional levels depending on the PIWI proteins. However, these mechanisms tolerate a low level of TE transposition which serves as both a driver of evolutionary processes and a source for basal piRNA biogenesis. Notably, piRNAs are mainly encoded by TE sequences localized in specific genomic regions forming arrays known as piRNA clusters (Brennecke et al., 2007; Gunawardane et al., 2007). These clusters, sometimes referred to as a genomic immune system, are classified as either uni-strand or dual-strand clusters, depending on whether they transcribe from one or both DNA strands.

**FIGURE 1:**
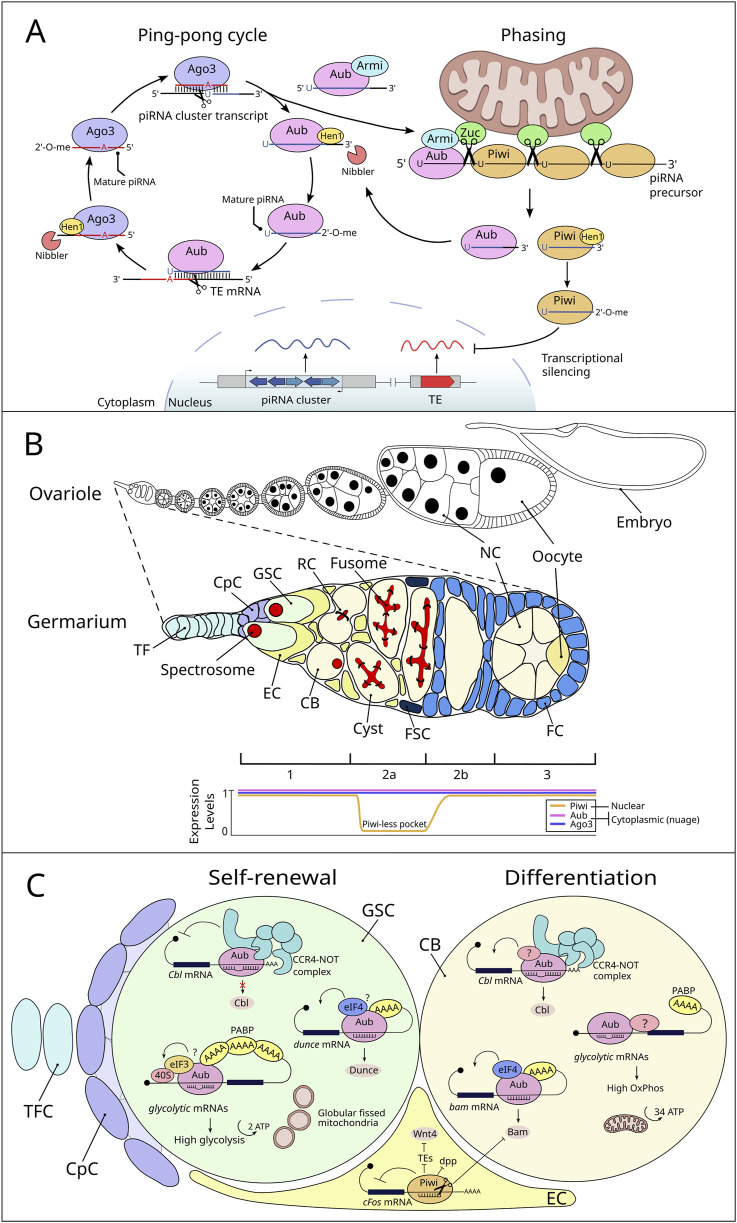
piRNA biogenesis in *Drosophila* germline and mRNA regulation by PIWI proteins and piRNAs in the GSC niche. **(A)** piRNA biogenesis in *Drosophila* germline. The ping-pong cycle occurs in the nuage and involves reciprocal cleavage of transposon mRNAs and piRNA cluster transcripts by two PIWI proteins, Aubergine (Aub) and Argonaute 3 (Ago3). Aub, guided by antisense primary piRNAs, cleaves transposon mRNAs to generate the 5′ ends of sense secondary piRNAs. Ago3-bound secondary piRNAs then cleave piRNA cluster precursors, producing 5′ ends of new primary piRNAs. The 3′ ends of pre-piRNAs are processed by the exonuclease Nibbler and modified with 2′-O-methylation by the methyltransferase Hen1, protecting them from degradation and resulting in mature piRNAs. In phased biogenesis, long piRNA precursors bound to Aub are transported by the RNA helicase Armitage (Armi) to the mitochondrial outer membrane. There, the endonuclease Zucchini (Zuc) cleaves the precursor, generating intermediate piRNAs with a characteristic 5′ uridine bias. The first piRNA returns to the nuage, while subsequent cleavages by Zuc create a series of Piwi-bound piRNAs. These are methylated by Hen1 and transported into the nucleus for transposon silencing. The choice between phasing and ping-pong processing is governed by the availability of substrates produced through secondary piRNA-guided cleavages and the regulatory role of Armi, which couples the two pathways despite their physical separation. **(B)** Schematic representation of a *Drosophila* ovariole and germarium with associated PIWI protein expression patterns. The top section depicts a *Drosophila* ovariole, showcasing the sequential stages of oogenesis. A zoomed-in view of the germarium is depicted below, detailing its cellular components (TF: terminal filament, CpC: cap cell, GSC: germline stem cell, EC: escort cell, CB: cystoblast, FC: follicle cell, FSC: follicle stem cell, NC: nurse cell). The bottom panel shows the expression pattern of the *Drosophila* PIWI protein family members (Piwi, Aub, and Ago3) in the germarium. These patterns highlight the “Piwi-less pocket” in region 2a, where Piwi expression is absent (Dufourt et al., 2014), while Aub and Ago3 are expressed across regions 1, 2a, 2b, and 3 and latter oogenesis. **(C)** Roles of PIWI/piRNAs in GSC maintenance and differentiation by gene expression regulation. Aub/piRNA complex regulates GSC self-renewal and differentiation by repressing *Cbl* mRNA through the CCR4-NOT complex, a repression reduced in cystoblasts (CBs), leading to higher Cbl levels. Additionally, Aub positively controls *dunce* and *bam* mRNAs in GSCs and CBs, respectively. Aub also influences the metabolic state of GSCs. Directed by piRNAs, Aub associates with glycolytic mRNAs to promote their translation in GSCs, resulting in increased glycolytic enzyme production. This mechanism likely involves Aub interacting with translation initiation factors such as PABP and eIF3. During differentiation, mitochondria undergo maturation, becoming more fused and structured with developed cristae, facilitating a metabolic shift towards oxidative phosphorylation. Other unidentified components (indicated by a question mark) may work alongside Aub to restrict glycolytic enhancement specifically to GSCs. In escort cells, Piwi plays a crucial role in regulating key signaling pathways and maintaining cellular functions. It suppresses Dpp signaling through an-as-yet-unknown mechanism. Piwi also targets *cFos mRNA* in the cytoplasm of cap and escort cells by binding to its 3′UTR, which leads to its cleavage into piRNAs and subsequent degradation of the *cFos* transcript. Furthermore, Piwi influences the expression of Wnt4 in escort cells by silencing transposable elements (TEs) at the transcriptional level.

Even though their differences, all piRNA clusters generate long precursor transcripts, which are transported to electro-dense cytoplasmic perinuclear foci. These foci are called Yb-bodies in gonadal somatic cells and nuage in germline cells in *Drosophila* (Huang et al., 2017). Within the nuage, piRNAs are amplified through a mechanism called the “ping-pong” cycle. Briefly, the piRNA precursors are cleaved to produce a first round of piRNAs. These primary piRNAs, guided by sequence complementarity, target to TE mRNAs and, through the endonucleolytic activity of PIWI proteins, cut the TE mRNAs precisely 10 nucleotides upstream of the 5′-end of the guide piRNA. This cleavage generates the 5′-end of a secondary piRNA on the opposite strand. A distinctive hallmark of ping-pong piRNAs is the presence of a 10-nucleotide overlap at their 5′-ends, with Aub-loaded piRNAs typically starting with an Uracil and Ago3-loaded piRNAs displaying an Adenine at position 10 from the 5′-end (Brennecke et al., 2007; Gunawardane et al., 2007; Nishida et al., 2007). In addition to the ping-pong cycle, an alternative piRNA biogenesis mechanism occurs at the outer mitochondrial membrane in both gonadal somatic and germline cells. This process, known as “phasing,” involves the RNA helicase Armitage (Armi) and the endonuclease protein Zucchini (Zuc) (Han et al., 2015; Mohn et al., 2015). Briefly, Armi facilitates the transport of Aub-bound pre-piRNA to the outer mitochondrial membrane, where it is processed by Zuc to generate an initial piRNA. Zuc cleaves the piRNA precursor to define its 3′-end, while the Piwi protein processes the 5′-end. This produces phased piRNAs that are loaded onto Piwi protein and subsequently translocated to the nucleus, where it transcriptionally silences TEs (Figure 1A). Thus, although Piwi protein is primarily localized in the nucleus, where it transcriptionally silences TEs, a small portion may transiently reside in the cytoplasm and contribute to the phasing process, unlike Aub and Ago3, which are predominantly cytoplasmic. Through these complementary mechanisms, the piRNA pathway effectively safeguards genome integrity while maintaining a controlled level of transpositional activity to support evolutionary dynamics and piRNA production. The choice between phasing and ping-pong processing for Aub depends on piRNA-guided slicing and Armi availability. Phasing processing relies on cleavages from the ping-pong cycle, which Armi facilitates by shuttling precursors from the nuage to mitochondria. Armi’s role is crucial, as its disruption halts phased piRNA production (Ge et al., 2019). Also, depletion of Aub or Ago3 significantly reduces Piwi-bound piRNAs. The frequency of ping-pong cleavages further regulates substrate supply, linking both pathways despite their spatial separation (Senti et al., 2015; Wang et al., 2015; Chary and Hayashi, 2023).

Beyond their canonical role in silencing TEs in animal gonads, piRNAs and PIWI proteins have been increasingly recognized for their functional roles in regulating gene expression at both transcriptional and post-transcriptional levels across diverse biological systems (Rojas-Ríos and Simonelig, 2018; Wang et al., 2023) (Table 1). The first direct evidence of piRNA involvement in mRNA regulation was observed in the regulation of the maternal gene *nanos (nos)* during early *Drosophila* embryogenesis (Rouget et al., 2010). This study demonstrated that piRNAs derived from the TEs *roo* and *412* guide the PIWI proteins Aubergine (Aub) and Argonaute 3 (Ago3) to the 3′UTR of *nos* mRNA, leading to its degradation via CCR4-NOT-mediated deadenylation. Subsequently, this novel role of piRNAs in mRNA destabilization has been extended to hundreds of mRNAs, particularly during two critical developmental processes: the maternal-to-zygotic transition (when the zygotic genome becomes transcriptionally active) and mouse spermiogenesis (Gou et al., 2014; Barckmann et al., 2015). During these processes, the piRNA pathway plays an essential role in orchestrating large-scale mRNA decay. Interestingly, PIWI proteins loaded with piRNAs have also been implicated in stabilizing cellular mRNAs by promoting poly(A) tail elongation and enhancing translational initiation. They activate mRNA translation through imperfect base-pairing interactions between piRNAs and their target mRNAs (Vourekas et al., 2016; Dufourt et al., 2017; Dai et al., 2019; Ramat et al., 2020). Thus, the piRNA pathway demonstrates versatile regulatory functions in mRNA processing and stability, which are critical not only for the aforementioned developmental processes but also for others, such as sex determination and fertility (Gou et al., 2014; Kiuchi et al., 2014). In addition to these roles, piRNAs and PIWI proteins are crucial for gene expression regulation at both chromatin and mRNA levels in stem cell biology. This regulation influences key processes such as chromatin remodeling, transcriptional silencing, and post-transcriptional control of mRNAs. This mini review highlights the emerging roles of PIWI proteins and piRNAs in regulating gene expression at both transcriptional and post-transcriptional levels, with a particular focus on their contributions to stem cell biology in highly regenerative species and *D. melanogaster*.

**TABLE 1:** mRNAs regulated by piRNAs/PIWI proteins in germ and soma cells.

PIWI protein	piRNAs	mRNA target	Expression		References
			Germline	Somatic cells	
Fruit fly (*Drosophila melanogaster)*					
Piwi	*flamenco*-derived piRNAs	TE mRNAs (*gypsy*, *Idefix*, *ZAM*), *Fos*	Primordial Germ Cells (PGCs), Germline stem cells (GSCs)	Cap cells and folicle cells	Brennecke et al. (2007), Post et al. (2014), Klein et al. (2016)
Aub	TE-derived piRNAs	Maternal mRNAs, *nanos*, *Su(Ste)*, *vasa*, *Cbl*, *dunce*, *bam*, glycolytic mRNAs	PGC; Ovary – GSCs, cyst, posterior pole of stage 10 oocyte, nurse cells; Testis - GSCs, gonialblasts, spermatogonia, spermatocytes	Expressed in gut, detected by qPCR	Harris and Macdonald (2001), Brennecke et al. (2007), Nishida et al. (2007), Tang et al. (2023), Ma et al. (2017), Rojas-Ríos et al. (2024)
Ago3	TE-derived piRNAs	TE mRNAs	PGCs; Ovary, predominantly in germarium – GSCs, cyst, oocyte, nurse cells; Testis – GSCs, sonialblasts, spermatogonia, spermatocytes		Brennecke et al. (2007), Gunawardane et al. (2007), Nagao et al. (2010)
Nematode (*Caenorhabditis elegans)*					
PRG-1	21U-RNAs	CSR-1 targets; *xol-1*	Gonad – GSCs, mitotic/meiotic germ cells, oocytes		Wu et al. (2019)
Zebra fish (*Danio rerio)*					
ZIWI	Anti-sense piRNAs	TE mRNAs	PGCs; Ovary – oogonia, oocytes; Testis – spermatogonia, spermatocytes		Houwing et al. (2008)
ZILI	Sense piRNAs	TE mRNAs; Meiosis-related transcripts	PGC; Ovary – oogonia, oocytes; Testis – spermatogonia, spermatocytes, spermatids	Embryonic soma	Houwing et al. (2008), Sun et al. (2010)
Mouse (*Mus musculus)*					
MIWI	Pachytene, pseudogene and TE piRNAs	Psma8, Ppp1cb, Atr, Gfpt1, Mdc1 (spermatogenesis-related genes)	Testis –meiotic spermatocytes, round and elongating spermatids		Deng and Lin (2002), Reuter et al. (2011), Zhang et al. (2015)
MILI	Pre-pachytene piRNAs	LINE1 elements	Testis –prospermatogonia, spermatogonia, spermatocytes, round spermatids		Aravin et al. (2006), Carmell et al. (2007), Shoji et al. (2009), De Fazio et al. (2011)
MIWI2	Pre-pachytene piRNAs	LINE1 elements	Testis – GSCs		Aravin et al. (2006), Carmell et al. (2007), Shoji et al. (2009), De Fazio et al. (2011), Watanabe et al. (2018)
Planarian (*Schmidtea mediterranea*/*Dugesia japonica)*					
SMEDWI-1	TE-derived piRNAs	TE mRNAs, Djcalu, Djhistone h4	Neoblast	Somatic stem cells	Reddien et al. (2005), Rouhana et al. (2014)
SMEDWI-2	TE-derived piRNAs	TE mRNAs	Neoblast		Reddien et al. (2005)
SMEDWI-3	TE-derived piRNAs and cellular piRNAs	Djmcm2, Djhistone h4	Neoblast		Rouhana et al. (2014)
*Hydra*					
Hywi		TE-mRNAs; putative non-TE targets in the interstitial lineage involved in cell cycle regulation	Nematoblast; Interstitial stem cells	Endodermal and ectodermal stem cells; epitelial cells	Juliano et al. (2014), Lim et al. (2014)
Hyli		TE-mRNAs; putative non-TE targets in the interstitial lineage involved in cell cycle regulation	Nematoblast; Interstitial stem cells	Endodermal and ectodermal stem cells	Juliano et al. (2014), Lim et al. (2014)
*Xenopus laevis*/*X. tropicalis*					
Xiwi	Single-strand piRNA clusters	TE-mRNAs; Gene transcripts	Embryos stage 1–20; Ovary – Stage I - IV and mature oocytes; Testis		Lau et al. (2009), Wilczynska et al. (2009), Toombs et al. (2017)
Xili	Single-strand piRNA clusters	TE-mRNAs; Gene transcripts	Embryos Stage 1–42; Ovary – Stage I - IV and mature oocytes; Testis		Lau et al. (2009), Wilczynska et al. (2009), Toombs et al. (2017)
Silkworm (*Bomboryx mori)*					
Siwi	Fem-derived piRNAs	*Masc* mRNA	Embryo Larvae: High in testis, low in ovary. Pupal/Adult: Low in testis, high in ovary		Kawaoka et al. (2008), 2011; Swevers et al. (2011), Kiuchi et al. (2014)
BmAgo3	Fem-derived piRNAs		Embryo Larvae: High in testis, low in ovary. Pupal/Adult: Low in testis, high in ovary		Kawaoka et al. (2008), 2011; Swevers et al. (2011), Kiuchi et al. (2014)

Summary of PIWI-family proteins, their interacting piRNAs, mRNA targets and cellular expression across model organisms.

### PIWI proteins and piRNAs in somatic stem cells of highly regenerative species

A diverse range of studies indicates that PIWI proteins are specifically expressed and required in somatic stem cells to support the regenerative capacity of several species, including sponges, acoels, cnidarians and planaria (Reddien et al., 2005; Palakodeti et al., 2008; Krishna et al., 2013; Rinkevich et al., 2013; Juliano et al., 2014; Lim et al., 2014; Ross et al., 2014; Bradshaw et al., 2015). In addition, piRNAs are expressed in the soma of *Hydra, Nematostella,* and the jellyfish *Sanderia malayensis* (Juliano et al., 2014; Praher et al., 2017; Nong et al., 2020). In planarian, PIWI proteins are expressed in somatic stem cells (known as neoblasts), and the loss of function of specific *piwi* genes compromises the ability of these animals to regenerate body parts due to defects in neoblast maintenance. Moreover, small RNAs produced by PIWI proteins are present in planarian soma cells, although only a small portion are complementary to TEs (Resch and Palakodeti, 2012; Shibata et al., 2016).

The functional role of PIWI and piRNAs in planarians involves silencing TEs as well as protein-coding genes. Notably, the nuclear PIWI protein regulates the expression of essential functional genes, such as *Djmcm2*, *Djhistone h4*, and *Djcalu*, which are involved in neoblast self-renewal and differentiation (Kashima et al., 2018). Interestingly, another study on planarian PIWI proteins, specifically the cytoplasmic SMEDWI-1 and SMEDWI-3, demonstrated their role in the localization of *histone H4* mRNA to chromatoid bodies in stem cells (Rouhana et al., 2014) suggesting distinct regulatory mechanisms employed by PIWI proteins for specific functional genes. Additionally, the nuclear PIWI plays a key role in TE silencing and regulates neoblast differentiation during cell specialization in the planarian *Dugesia japonica* (Shibata et al., 2016). An independent study revealed that SMEDWI-3 has a dual role in mRNA turnover in planarian neoblasts (Kim et al., 2019). It degrades certain mRNAs through a homotypic ping-pong cycle while binding to others, guided by antisense piRNAs, without causing degradation. These distinct functions are determined by the level of complementarity between the target mRNAs and antisense piRNAs, highlighting the critical regulation of neoblast mRNA turnover in planarians by piRNAs. Furthermore, a recent study showed that the planarian PIWI protein SMEDWI-2 is crucial for guiding stem cells through chromatin transitions during differentiation in the planarian *Schmidtea mediterranea* (Li et al., 2021). Overall, PIWI proteins and their associated piRNAs are integral to somatic stem cell function and regenerative processes suggesting that this mechanism is a conserved feature through evolution.

### Non-cell-autonomous function of PIWI and piRNAs in GSC self-renewal and differentiation

Like its role in silencing TEs, the regulation of gene expression by the piRNA pathway has been extensively studied in the GSCs of the *Drosophila* female. The *Drosophila* ovary provides an exceptional model for investigating stem cell regulation *in vivo* (Rojas-Ríos and González-Reyes, 2014; Rosales-Nieves and González-Reyes, 2014). Each ovary consists of 16–20 ovarioles, each composed of an anterior germarium that transitions into progressively maturing follicles (Figure 1B). Within the germarium, two to three GSCs reside in a somatic cellular niche of three distinct somatic cell types: terminal filament cells (TFCs), cap cells (CpCs), and escort cells (ECs) (Losick et al., 2011; Jin and Zhao, 2023). The GSCs divide asymmetrically inside the niche to give rise to a cystoblast (CB) that undergoes four rounds of synchronous divisions to generate a 16-cell germline cyst that will ultimately produce a mature oocyte. Within the GSCs themselves, spectrosomes-dynamic membranous structures- undergo shape changes throughout the cell cycle and are critical for proper mitotic spindle orientation (Villa-Fombuena et al., 2021; Sánchez-Gómez et al., 2024). This ensures the asymmetric division necessary to maintain the stem cell pool while producing differentiating daughter cells. GSCs are anchored to the niche via E-cadherin and adherens junctions, effectively maintaining their self-renewal capacity and preventing their migration outside the niche (Dansereau and Lasko, 2008). The main signaling system from niche cells is Decapentaplegic (Dpp), a bone morphogenetic protein (BMP) ligand that promotes GSC self-renewal within a short range (Harris and Ashe, 2011). Dpp signaling represses *bam* expression specifically within GSCs, essential for GSC differentiation since Bam is required and sufficient for germline differentiation (Xie and Spradling, 1998). This repression is relieved once a GSC daughter leaves niche since the movement and stability of Dpp is restricted in the niche by Collagen IV and Glypican Dally, a protein whose expression is controlled by the EGFR-MAPK signaling pathway (Wang X. et al., 2008; Guo and Wang, 2009; Hayashi et al., 2009; Liu et al., 2010). The expression of Dpp in the GSC niche is highly controlled by different mechanisms including the JAK-STAT, Hedgehog and Piwi pathways (López-Onieva et al., 2008; Wang L. et al., 2008; Rojas-Ríos et al., 2012; Jin et al., 2013).

Early studies using *piwi* mutants demonstrated that *piwi* regulates GSC self-renewal and differentiation (Cox et al., 1998; 2000; Szakmary et al., 2005). Further studies of the role of *piwi* in GSC biology revealed that its cell-autonomous function regulates GSC self-renewal and asymmetric division, while its non-cell-autonomous function in the niche is essential for early germline differentiation. Specifically, *piwi* acts in ECs to promote GSC differentiation by regulating several signaling pathways (Figure 1C). Genetic analyses indicate that *piwi* represses *dpp* expression in ECs to limit its diffusion, thereby promoting germline differentiation (Jin et al., 2013). However, *dpp* is not the only factor regulated by *piwi* in ECs to maintain GSCs and support differentiation. A noteworthy study demonstrated that *piwi* represses the *c-Fos* proto-oncogene at the mRNA level in ECs. Importantly, this post-transcriptional repression occurs through the 3′UTR of *c-Fos* mRNA, leading to its cleavage and the production of piRNAs. This finding indicates that cellular *c-Fos* mRNA serves as a source for piRNA biogenesis in somatic cells of the gonad (Klein et al., 2016). Additionally, *Piwi* control of TEs in ECs plays a critical role in repressing *Wnt4* expression, a key signal involved in germline differentiation and cystoblast encapsulation (Upadhyay et al., 2016). Specifically, mutations in soma piRNA pathway components, such as *piwi* and *flamenco* (but not *aubergine*), were shown to reduce *Wnt4* expression, as demonstrated by qRT-PCR and *in situ* hybridization. These mutants also exhibited germline differentiation defects, further underscoring the importance of *Wnt4* regulation. These findings suggest that *Piwi* promotes GSC differentiation by repressing *Wnt4* expression in ECs through its TE control function (Figure 1C). By maintaining TE silencing, *Piwi* ensures proper signaling in the somatic niche, allowing for the differentiation of GSCs and the proper encapsulation of CBs, highlighting its pivotal role in regulating the balance between stem cell self-renewal and differentiation. A novel identified role of Piwi in the GSC niche involves maintaining GSC adhesion to CpCs. Like its function in ECs, Piwi silences TEs to prevent the activation of Toll-GSK3 signaling, which would otherwise lead to the degradation of β-catenin (a critical component of the Cadherin-Catenin-Actin complex that mediates cell adhesion). Importantly, this recent study demonstrated that aging CpCs express reduced levels of Piwi. This decline results in TE-dependent activation of the Toll receptor through an unknown mechanism, disrupting β-catenin stability. Consequently, reduced Piwi levels in aged niches lead to GSC detachment from CpCs, impairing GSC self-renewal and contributing to age-associated niche deterioration. This highlights the essential role of Piwi in preserving the structural integrity and functionality of the GSC niche during adulthood (Lin et al., 2020).

### Cell-autonomous functions of PIWI and piRNAs in GSC self-renewal

An epigenetic mechanism involving Piwi has been identified as essential for GSC maintenance in female *Drosophila* (Peng et al., 2016). A genome-wide screen designed to identify suppressors of *piwi* uncovered a partner protein associated with Polycomb group (PcG) proteins (Smulders-Srinivasan and Lin, 2003). PcG proteins are crucial epigenetic regulators that modulate chromatin through histone methylation, particularly by adding H3K27me3 marks. These marks are generally associated with transcriptional repression, while reduced H3K27me3 levels correlate with active transcription mediated by RNA polymerase II. Piwi interacts with the Polycomb Repressive Complex 2 (PRC2) in the nucleoplasm, playing a key role in regulating chromatin state. This interaction inhibits PRC2 binding to genomic regions that do not directly interact with Piwi, resulting in reduced H3K27me3 levels and altered transcriptional activity, which are critical for oogenesis and GSC maintenance. Piwi appears to modulate RNA polymerase II function by sequestering PRC2 in the nucleoplasm, thereby restricting its access to genomic targets and preventing excessive transcriptional repression. These findings suggest a dual role for Piwi: modulating chromatin states in both niche cells and GSCs to create a favorable transcriptional environment for self-renewal and differentiation.

Aub, another *Drosophila* PIWI protein, plays a crucial role in gene expression regulation through direct binding to specific mRNAs in GSCs. Recently, it has been described that Aub binds mRNAs encoding glycolytic enzymes like *Enolase (Eno)*, to promote their translational activation. This process is guided by piRNAs, which enable Aub to interact with regions of target mRNAs, such as untranslated regions (UTRs). Mutations in the piRNA-binding sites within the *Eno* 5′UTR result in reduced *Eno* expression and subsequent GSC loss, underscoring the importance of precise mRNA regulation in maintaining glycolytic flux and GSC self-renewal (Rojas-Ríos et al., 2024). High glycolytic activity is essential for GSC maintenance, while disruptions in Aub function led to a metabolic shift towards oxidative phosphorylation (oxphos), characterized by reduced glycolytic enzyme levels, increased ATP synthase expression, and premature mitochondrial maturation in GSCs. These metabolic changes are incompatible with the maintenance of GSCs, as increasing glycolysis via Phosphofructokinase overexpression partially rescues GSC loss in *aub* mutants. In addition to glycolytic mRNAs, Aub regulates a broader spectrum of transcripts critical for GSC fate transitions. For instance, Aub represses *Cbl* mRNA to support GSC self-renewal by recruiting the CCR4-NOT deadenylation complex, which is also required for maintaining GSC self-renewal (Joly et al., 2013). Notably, this repression occurs without poly(A) tail shortening, suggesting an alternative mechanism of translational inhibition (Rojas-Ríos et al., 2017). Conversely, Aub also activates *dunce* mRNA translation, further illustrating its capacity for dual regulatory roles depending on the specific target and cellular context (Ma et al., 2017). Additionally, PIWI proteins are capable of positively regulate target mRNAs under certain conditions. For example, Aub promotes the translation of mRNAs like *bam* and *dunce* at precise stages of the GSC lineage, ensuring precise temporal controls over developmental processes. These findings illustrate that the piRNA-PIWI pathway employs a context-dependent mechanism to either repress or activate mRNA targets, thereby maintaining GSC properties such as self-renewal and differentiation.

### Piwi in intestinal stem cells of *Drosophila*

Recent research has explored the role of Piwi in regulating intestinal homeostasis in *Drosophila* (Tang et al., 2023). Piwi expression has been detected in the adult gut at both mRNA and protein levels, as confirmed by RT-PCR and Western blot analyses. Additionally, Piwi-Gal4-driven GFP expression reveals that intestinal stem cells (ISCs) and gut progenitors express Piwi specifically. Immunostaining analysis further shows that Piwi protein is localized in the cytoplasm of ISCs, suggesting a potential role in post-transcriptional regulation. As Piwi protein is primarily nuclear in the gonads, and the analysis lacks an antibody specificity control for the gut, the claim regarding Piwi’s cytoplasmic localization should be interpreted with caution. Nevertheless, to identify Piwi’s target genes in the gut, mRNA sequencing of *piwi* mutant guts reveals hundreds of dysregulated protein-coding genes (Tang et al., 2023). Gene ontology analysis indicates that several metabolic processes, including carbohydrate metabolism and reactive oxygen species (ROS) response pathways, are affected. Given that ROS levels impact various stem cell populations, Piwi may play a critical role in maintaining stem cell homeostasis through ROS regulation. Notably, Piwi’s involvement in ISC maintenance appears to influence adult longevity. Other Argonaute family members, such as Aub, Ago3, and Ago2, also impact lifespan, suggesting a broader role for silencing pathways in adult survival. Surprisingly, small RNA sequencing fails to detect piRNAs in the gut, a finding confirmed by an independent study (Siudeja et al., 2021), suggesting a piRNA-independent role in the gut for PIWI proteins. Additionally, this work identified TE insertions in the tumor suppressor gene Notch within *Drosophila* ISCs, which may contribute to the development of gut neoplasia (Siudeja et al., 2021). Moreover, Piwi function has been examined under acute proliferative conditions, such as cancer development or enteropathogenic infection (Sousa-Victor et al., 2017). In these contexts, Jak/STAT-dependent Piwi activation in ISCs is essential for the proliferative response, TE silencing, genome integrity, and apoptosis suppression. Together, these studies underscore the essential role of Piwi in somatic stem cell regulation under both physiological and pathological conditions, highlighting its involvement in broader molecular mechanisms such as TE repression and cellular metabolism.

## Discussion

Stem cells, characterized by their capacity for self-renewal and differentiation into specialized cell types, play a fundamental role in development and tissue maintenance throughout life. A notable feature of many stem cells populations is their reliance on glycolysis over mitochondrial oxphos. This preference is reminiscent of the Warburg effect in cancer cells, where high glycolytic activity supports rapid proliferation (Liberti and Locasale, 2016; Liu and Chen, 2021). However, the exact functional significance of elevated glycolysis in stem cells remains incompletely understood.

Emerging evidence suggests that glycolytic enzymes may play roles beyond metabolism, functioning as RNA-binding proteins (RBPs) that regulate post-transcriptional gene expression (Castello et al., 2012). Various metabolic enzymes, including glycolytic enzymes such as Aldolase, Enolase, Hexokinase and Pyruvate Kinase, have been identified as RBPs (Baltz et al., 2012). Recent studies underscore the multifunctionality of these enzymes. For example, Enolase was shown to regulate embryonic stem cell differentiation through riboregulation of specific mRNAs (Huppertz et al., 2022), while Hexokinase demonstrated a nuclear role in hematopoietic stem cell maintenance by modulating chromatin accessibility and DNA integrity (Thomas et al., 2022). Moreover, in *Drosophila*, glycolytic enzymes have been implicated in piRNA biogenesis by binding Tudor proteins (Gao et al., 2015). Additionally, research has revealed that glycolytic enzymes are essential for the maintenance and function of GSCs in *Drosophila* (Rojas-Ríos et al., 2024). Specifically, Ald, Eno, and Pyk are expressed at higher levels in GSCs compared to differentiated germline cells and are required for both GSC maintenance and piRNA biogenesis (Gao et al., 2015; Rojas-Ríos et al., 2024). Importantly, Aub regulation of glycolytic mRNAs establishes a direct connection between translational control, metabolic programming, and piRNAs in stem cell biology. Furthermore, the mechanisms by which PIWI proteins regulate mRNAs appear to be evolutionarily conserved across species. For instance, in mammalian systems, homologs such as Miwi similarly activate translation by interacting with poly(A)-binding proteins and eIF3 subunits (Dai et al., 2019; Ramat et al., 2020). Overall, these parallels suggest that the PIWI-mediated regulation of cellular mRNA translation is a fundamental feature of developmental biology. Since the expression of PIWI proteins and piRNAs, along with elevated glycolysis, are hallmark features of both stem cells and cancer cells, it would be highly interesting to investigate whether the piRNA pathway regulates energy metabolism in cancer cells and whether glycolytic enzymes perform moonlighting functions as RBPs in these cell types.
